# Complex Regulatory Networks Governing Production of the Glycopeptide A40926

**DOI:** 10.3390/antibiotics7020030

**Published:** 2018-04-05

**Authors:** Rosa Alduina, Margherita Sosio, Stefano Donadio

**Affiliations:** 1 Department of Biological, Chemical and Pharmaceutical Sciences and Technologies (STEBICEF), Università degli Studi di Palermo, Viale delle Scienze Bd.16, 90128 Palermo, Italy; 2 Via Ortles 22/4, Naicons Srl, 20139 Milano, Italy; msosio@naicons.com (M.S.); sdonadio@naicons.com (S.D.); 3 Via Ortles 22/4, Ktedogen Srl, 20139 Milano, Italy

**Keywords:** glycopeptide antibiotics, *dbv* cluster, regulatory genes, StrR, LAL, LuxR solo, dalbavancin, A40926

## Abstract

Glycopeptides (GPAs) are an important class of antibiotics, with vancomycin and teicoplanin being used in the last 40 years as drugs of last resort to treat infections caused by Gram-positive pathogens, including methicillin-resistant *Staphylococcus aureus*. A few new GPAs have since reached the market. One of them is dalbavancin, a derivative of A40926 produced by the actinomycete *Nonomuraea* sp. ATCC 39727, recently classified as *N. gerenzanensis*. This review summarizes what we currently know on the multilevel regulatory processes governing production of the glycopeptide A40926 and the different approaches used to increase antibiotic yields. Some nutrients, e.g., valine, l-glutamine and maltodextrin, and some endogenous proteins, e.g., Dbv3, Dbv4 and RpoB^R^, have a positive role on A40926 biosynthesis, while other factors, e.g., phosphate, ammonium and Dbv23, have a negative effect. Overall, the results available so far point to a complex regulatory network controlling A40926 in the native producing strain.

## 1. The Glycopeptides

The glycopeptides are a class of antibiotics with a complex chemical structure and relatively high molecular weight. Since 1953, about 50 glycopeptide antibiotics (GPA) have been isolated [1], and several of these have been approved for clinical use. These include vancomycin, produced by *Amycolatopsis orientalis* and marketed in 1958, and teicoplanin, produced by *Actinoplanes teichomyceticus* and marketed in 1987. The second-generation glycopeptides telavancin, derived from vancomycin, dalbavancin, derived from A40926, and oritavancin, derived from choloroeremomycin, were introduced onto the market in 2009, 2014 and 2015, respectively. All glycopeptides are used to treat persistent infections by Gram-positive multi-resistant pathogens [2]. The second-generation glycopeptides are nearly 4- to 8-fold more effective than vancomycin against Gram-positive pathogens, and are also active against vancomycin-intermediate or vancomycin-resistant strains of *Staphylococcus* and *Enterococcus* spp. [3]. While dalbavancin impedes the late steps of cell wall biosynthesis principally by blocking transglycosylase activity, oritavancin and telavancin bind to the bacterial membrane by the lipophilic side chain linked to their disaccharide moiety, disturbing membrane integrity and leading to bacteriolysis [3].

Chemically, glycopeptides are a class of molecules constituted by a heptapeptide core consisting of both proteinogenic and non-proteinogenic amino acids, such as 3,5-dihydroxyphenylglycine (Dpg) and 4-hydroxyphenylglycine (Hpg). A heptapeptide is produced by a non-ribosomal peptide synthetase (NRPS) and, while tethered to the large multi-functional enzyme, the peptide scaffold is made rigid through oxidative cross-linking of the electron-rich aromatic side chains by P450s and chlorinated [4,5,6]. Further tailoring steps may include one or more glycosylations, methylation, sulfation and modification of the added sugar(s) by acylation and acetylation.

Glycopeptide producers are widespread among distantly related genera of actinomycetes [1]: vancomycin, balhymicin and ristocetin were isolated from distinct species of the genus *Amycolatopsis*, belonging to the family *Pseudonocardiaceae*; teicoplanin and UK-68597 are produced by members of the genus *Actinoplanes*, family *Micromonosporaceae*; A40926 is from the genus *Nonomuraea*, family *Streptosporangiaceae*; and pekiskomycin and A47394 are from the genus *Streptomyces*, family *Streptomycetaceae* [7]. Thus, production of GPAs is widespread among actinomycetes, as shown by the relatively high frequency at which glycopeptide producers can be detected in environmental samples after applying appropriate selection procedures [8].

The medical interest and importance of these molecules has prompted the analysis of the genes required for their synthesis. Different glycopeptide biosynthetic gene clusters have been reported [9]; combining the information obtained from these clusters, a function has been assigned to most genes involved in glycopeptide formation by in vivo gene disruption in the producing strain(s) and by biochemical studies of the overproduced enzymes. While these results have analyzed different pathways, the emerging overall picture has contributed to deciphering most of the biosynthetic steps and the timing of the events in the biosynthesis of all GPAs [4,5,6,8,10].

## 2. Development of Dalbavancin

**Figure 1:**
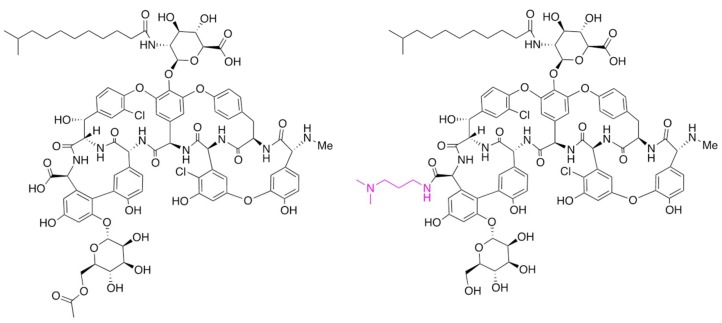
Chemical structures of *O*-acetyl A40926 and of dalbavancin. Only the component B0 is shown for simplicity. The chemical modification present in dalbavancin is indicated in red type.

Dalbavancin is a second-generation glycopeptide derived from A40926 with an improved antibacterial activity over teicoplanin, the most closely correlated marketed GPA. The enhanced pharmaco-dynamic properties of the molecule and lipophilic anchoring to the bacterial cell membrane confer more potent in vitro and in vivo activity than teicoplanin. The most prominent peculiarity of dalbavancin is a significantly extended half-life in plasma, which allows once-a-week dosing by intravenous injection. The drug has been approved for treating complicated acute bacterial skin and skin structure infections. Its synthesis involves the deacetylation of the final biosynthetic intermediate A40926 (a process achieved during recovery from the fermentation broth), protection of the carboxyl group present in the aminosugar, conversion of the C-terminal carboxyl group into a (3-dimethylamino)-1-propylamide, and final deprotection of the aminosugar carboxyl group [11]. The main components of the A40926 complex differ mainly in the acyl chain attached to the sugar, with B_0_ and B_1_ as the major representatives, characterized respectively by an iso-C12:0 and a n-C12:0 acyl moiety bound to the aminoglucuronic acid moiety [12]. The structures of A40926 and of dalbavancin are shown in Figure 1.

Dalbavancin obtained market authorization in 2014 in the USA and the following year in Europe. This was a noteworthy success in view of the intricate history related to its development, which started back in the early 1990s and involved at least six different legal entities, as recently summarized [13]. 

**Figure 2:**
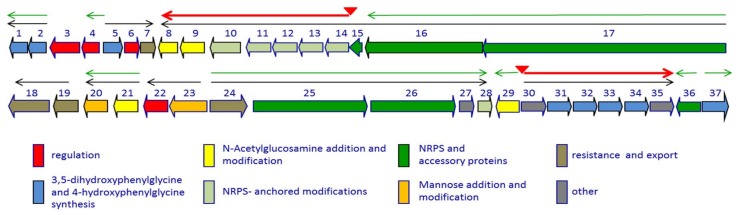
Genetic organization of the *dbv* cluster. The thin black arrows indicate experimentally determined operons. Red triangles indicate experimentally determined Dbv4 binding sites, with the corresponding transcripts as red thick arrows; the thin green arrows represent the transcriptional units controlled by Dbv3. The *dbv* genes are grouped by functional category as indicated. See also Table 1.

**Figure 3:**
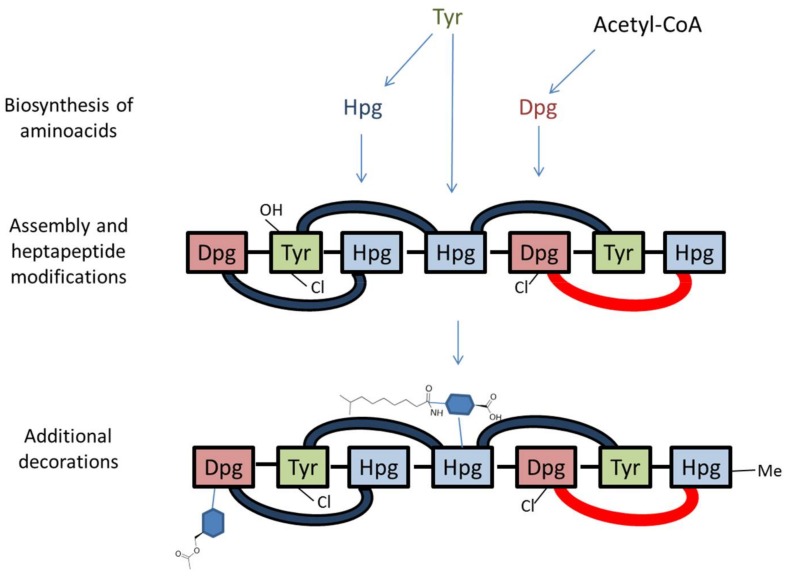
Simplified model of *O*-acetyl A40926 biosynthesis. Note that the heptapeptide is drawn right (N-terminus) to left (C-terminus), consistent with Figure 1. Cross-links are indicated by blue (C–O–C) or red (C–C) arcs. Sugars are represented as blue hexagons. Refer to Figure 2 and Table 1 for details.

In this review, we have organized the text into three separate sections: the first concerns the improvement of antibiotic yield by modifying the media components; the second describes the biosynthetic gene cluster and its transcriptional organization (Figure 2), along with the biosynthetic steps (Figure 3); and the last section deals with the cluster specific regulatory genes.

## 3. Improvement of A40926 Production

Improvement of glycopeptide production has very likely been achieved through several rounds of mutagenesis and screening, leading to the current industrial strains producing vancomycin, teicoplanin, chloroeremomycin and A40926. However, most of this work has not surfaced in the scientific literature, and we will limit ourselves to published reports on the A40926 process. 

**Figure 4:**
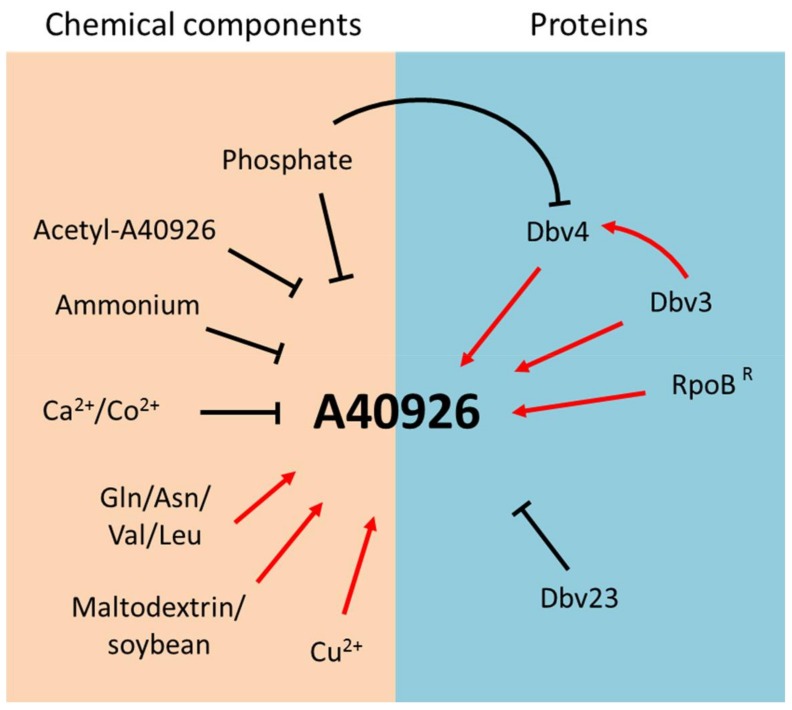
Nutrients, biosynthetic products and proteins regulating A40926 production in *Nonomuraea gerenzanensis*.

Initial work established the influence of growth conditions on A40926 production by *Nonomuraea* sp. ATCC 39727, recently classified as *N. gerenzanensis* [14]. In a chemically defined medium, low initial concentrations of phosphate and ammonium led to increased A40926 production, while glucose limitation did not (Figure 4). In particular, the level of residual ammonium and phosphate strongly influenced A40926 production rates and final titers, but not the initiation of production [15]. In a similar medium, A40926 production was repressed by calcium, but supported when l-glutamine or l-asparagine were added as nitrogen sources instead of ammonium salts (Figure 4) [16]. Since the catabolic products of branched chain amino acids represent biosynthetic precursors for the formation of the branched chain acyl moieties of A40926 [17], studies were undertaken on the influence of valine supplementation. Addition of 1 to 3 g/l-valine to complex media improved both the relative and absolute production of the B_0_ congener with decrease of the B_1_ component in the A40926 complex [18]. A40926 yields were found to also be controlled by stringent response in both complex and chemically defined media (Figure 4) [19]. 

It has also been recently reported that a *Nonomuraea* strain producing high levels of A40926 in an optimized production medium was isolated after UV mutagenesis. This mutant strain was used to study the effect of carbon and nitrogen sources and of different ions on antibiotic productivity; addition of the scarcely assimilated carbon source maltodextrin and the nitrogen source soybean meal strongly affected A40926 production, which reached 1 g/L in a 10-L fermenter. Furthermore, Cu^2+^ stimulated A40926 biosynthesis while Co^2+^ showed an inhibitory effect. As shown for valine, even l-leucine addition led to an increased production of total A40926 and changed the complex toward the B_0_ compound (Figure 4) [20]. While the shift in complex composition after amino acid addition can be easily rationalized, there are currently no clues as to why certain carbon sources and metal ions stimulate or inhibit growth and/or A40926 production.

## 4. The *dbv* Gene Cluster: Main Features

The characterization of the gene cluster necessary for A40926 biosynthesis [21] laid the foundation for understanding the regulatory mechanisms working in the producer strain [22,23]. The *dbv* gene cluster is constituted by 37 protein coding sequences involved in antibiotic biosynthesis, regulation, immunity, and export [21] (Figure 2). 

In particular, Dbv1, Dbv2, Dbv5, Dbv30-34 and Dbv37 are involved in biosynthesis of the two non proteinogenic amino acids Hpg and Dpg, while Dbv16-17 and Dbv25-26 constitute the NRPS that joins the amino acids Hpg, Tyr, Dpg, Hpg, Hpg, Tyr and Dpg in a ribosome-independent manner. The A40926 aryl groups are linked by three ether links and one C–C link through the action of Dbv11-14 P450s, while the single halogenase Dbv10 chlorinates Dpg-3 and Tyr-6. By analogy with other glycopeptides, halogenation should occur on an NRPS-bound substrate [5], while Tyr beta hydroxylation [24] might also involve interaction with an NRPS-bound substrate or intermediate. Additional modifications require the action of: Dbv27, for *N*-methylation of the terminal Hpg-1 residue; Dbv9, Dbv21, Dbv8 and Dbv29, for addition of *N*-acetyl glucosamine, deacetylation and acylation with long chain fatty acids, and sugar oxidation, respectively [25,26,27]; and Dbv20 and Dbv23, for mannosylation of Dpg-7 and its *O*-acetylation [28]. The different functions are illustrated in Figure 2 and summarized in Table 1, and a simplified model of *O*-acetyl A40926 biosynthesis is depicted in Figure 3. 

The last biosynthetic step is possibly represented by acetylation at position 6 of the mannose moiety carried out by Dbv23 [28,29]. A strain deleted in *dbv23* produced only glycopeptides lacking the O-linked acetyl residue. Interestingly, antibiotic production in a complex medium by the mutant strain occurred at twice the levels of the wild type. The low amount of glycopeptide produced by the wild-type strain might be dependent upon an inhibitory effect exerted by the acetylated compound, the final pathway intermediate. Consistently, spiking the production medium with 1 µg/mL of the acetylated glycopeptide inhibited total glycopeptide production in the mutant strain, while the deacetylated glycopeptide had no effect [28]. It is thus tempting to speculate that A40926 production is regulated by its end product, ensuring that A40926 does not occur during growth of the strain. This might occur through a two-component signal transduction process, in which a specific receptor could activate a response regulator and repress A40926 biosynthesis. This might be relevant in industrial processes, in which a seed culture is eventually used to inoculate the production medium. Any A40926 produced in the seed culture might be sufficient to inhibit A40926 production when the strain is inoculated in the production medium. This mechanism might be related to the inherent sensitivity of the strain to its own product, as described below.

Glycopeptides bind to the D-Ala-D-Ala portion of lipid II and thus inhibit the transpeptidation and transglycosylation reactions, thereby blocking peptidoglycan polymerization. The first and best characterized mechanism for glycopeptide resistance was established in enterococci, where glycopeptide action is avoided by deploying a modified target through a complex process that requires at least three biosynthetic genes (*vanHAX*) and a regulatory circuit (reviewed in [30,31,32]). Glycopeptide resistance in actinomycetes can also involve reprogramming of the peptidoglycan precursor by the action of VanHAX-related enzymes, as, for example, in *Amycolatopsis balhimycina* [33,34]. Instead, *Nonomuraea gerenzanensis* lacks the typical *vanHAX* cassette and the *dbv* cluster encodes the carboxypeptidase Dbv7, which has been shown to provide a modest but measurable resistance effect in the wild-type strain and in a heterologous background [35]. It should be noted that glycopeptide resistance in actinomycetes is still far from being completely understood, with there being a subtle interplay between glycopeptide resistance and glycopeptide tolerance [36]. Finally, the ABC transporters Dbv18, Dbv19, and Dbv24 and ion-dependent transmembrane transporter Dbv35 may contribute to glycopeptide resistance through active export from the cell, as observed for the Dbv24 homolog in the balhimycin producer [37]. 

The transcriptional organization of the *dbv* cluster was elucidated by RT-PCR targeting desired regions of the gene cluster [22]. The results, illustrated in Figure 2, denote a complex transcriptional organization, with at least 14 promoters, the two-gene operons *dbv1*-*dbv2*, *dbv19*-*dbv18*, *dbv21*-*dbv20*, *dbv23*-*dbv22*, the larger operons *dbv5*-*dbv7*, *dbv24-dbv28*, and *dbv30*-*dbv35*, and the largest operon *dbv17*-*dbv8*. Apparently, *dbv3*, *dbv4*, *dbv29*, *dbv36* and *dbv37* are transcribed as monocistronic units. The results are summarized in Table 1, which also lists the functions of the corresponding proteins. Real-time RT-PCR showed that a promoter is present upstream to *dbv14*, directing expression of the *dbv14-dbv8* operon through a leaderless transcript. However, a longer operon is likely to be transcribed from upstream promoter(s), since RT-PCR analysis showed the existence of a transcript spanning *dbv15* and *dbv14*. Some of the associated regulatory networks controlling A40926 biosynthesis are described below.

## 5. Cluster-Specific Regulatory Genes

The *dbv* cluster contains two regulatory genes, *dbv3* and *dbv4,* and the members of a putative two-component system, *dbv6* and *dbv22* [21,22,23]. Over a decade ago, a comparative analysis of the then-available five glycopeptide gene clusters—namely, those for chloroeremomycin, balhimycin, A47934, A40926 and teicoplanin—revealed that a StrR-like protein (i.e., Dbv4) was present in all clusters [38]. We previously demonstrated cross-binding among StrR-like regulators from glycopeptide clusters; specifically, Bbr (from the balhimycin cluster) can bind to the *dbv30* upstream region, while Dbv4 binds to the regions upstream of *bbr* and *oxyA* in the balhimycin cluster [22]. The target regions of Bbr and Dbv4 contain the highly conserved palindromic consensus sequence GTCCAR(N)_17_TTGGAC. This sequence was considered to be the Dbv4 binding site and was found in two regions of the *dbv* cluster and in five regions of the balhimycin cluster [39]. Consistently, this conserved palindrome is part of a conserved intergenic region present in the five glycopeptide clusters mentioned above [38]. 

In addition to the common regulation of the oxygenase transcription through a Dbv4-type regulator, diverse regulatory schemes are apparently used in the other biosynthetic gene clusters. Actually, in the teicoplanin cluster, Dbv4-like protein also positively regulates the transcription of the gene operon involved in Dpg biosynthesis and, as a matter of fact, the Dbv4 target sequence was found upstream of this operon, suggesting a Dbv4-type dependent regulation. In contrast, while Bbr, through the binding of its upstream region [39], functions as an autoregulatory protein, Dbv4 did not. Similarly, since the conserved palindrome is apparently missing in the region upstream of the corresponding genes in the A47394 and teicoplanin, the Dbv4-like regulator is not expected to control its own expression in these clusters. 

A40926 production is repressed by high initial concentrations of phosphate, and this repression was demonstrated to occur through Dbv4 [22]: phosphate depletion induces *dbv4* transcription in a defined medium, allowing Dbv4 to enhance expression of the operons *dbv14*-*dbv8* and *dbv30*-*dbv35*. However, phosphate did not influence the expression of most analyzed *dbv* genes [22]. The biosynthesis of many diverse secondary metabolites is controlled by phosphate [40]. Phosphate control of antibiotic biosynthesis in *Streptomyces coelicolor* and *S. lividans* is dependent upon the two-component system PhoR-PhoP [41], and PhoP was found to bind promoters of phosphate-regulated genes in *S. coelicolor* [42]. However, we were unable to identify Pho boxes in the region upstream of *dbv4* [22], suggesting divergence in phosphate control of antibiotic biosynthesis in different actinomycetes.

Another regulator from the *dbv* gene cluster has been experimentally characterized: Dbv3, a LuxR solo regulator belonging to the large ATP-binding regulators of the LuxR protein family. Dbv3 positively regulates A40926 production, since the ∆*dbv3* strain does not produce antibiotic and shows reduced transcription levels of *dbv4* and of many other *dbv* genes [23]. Thus, both the LuxR- and StrR-like regulators act as activators of A40926 biosynthesis.

The experimental evidence obtained for different glycopeptide pathways indicates that the StrR-like regulators Bbr, Tei15 and Dbv4 regulate balhimycin, teicoplanin and A40926 biosynthesis, respectively [22,39,43], whereas the LuxR-like regulators Dbv3 and Tei16 positively regulate A40926 and teicoplanin biosynthesis, respectively [23,43]. The balhimycin cluster does not encode a LuxR-like regulator. In A40926 biosynthesis, Dbv3 positively regulates Hpg biosynthesis, heptapeptide backbone biosynthesis, mannosylation, hexose oxidation and export. In addition, Dbv3 was found to hierarchically control *dbv*4 transcription in a cascade-like regulatory mechanism, so that Dpg biosynthesis and transcription of the *dbv14*-*dbv8* operon are also under indirect control of Dbv3. In addition, Dbv4 and Dbv3 expression seems to be differently modulated, since transcription of *dbv4* and Dbv4 target genes was found to be repressed by phosphate, while the Dbv3 target genes were not [22]. It should be noted that in teicoplanin biosynthesis, the expression of at least 17 genes is directly governed by Tei15, the Dbv4-like regulator, which directly controls transcription of *tei*16, the *luxR*-type regulator [43]. The targets of Tei16 have not been reported yet. 

Notwithstanding the absence of obvious targets for yield improvements by gene knockouts (e.g., repressor genes), genetic manipulation of selected *dbv* genes has led to increased yields of A40926. Knockout of the acetyltransferase *dbv23* (see above) or overexpression of Dbv3 resulted in higher (2-fold) A40926 production than in the wild type strain in rich medium, providing useful examples of knowledge-based strain improvement [23,28]. Analysis of the additional regulators encoded by the *dbv* cluster, the sensor kinase Dbv22 and the response regulator Dbv6, has established their role in the regulation of A40926 and provided additional strategies for rational intervention.

## 6. Future Perspectives

This review summarizes the main achievements in understanding A40926 biosynthesis in *N. gerenzanensis* in relation to other glycopeptide producers and model *Streptomyces* strains. While many studies have addressed antibiotic production in model streptomycetes, like *S. coelicolor*, we continuously learn new mechanisms and pathways as we extend these analyses to industrially relevant antibiotics and, especially, to actinomycetes other than *Streptomyces* spp. In this respect, strains belonging to the genus *Nonomuraea* represent complex systems, with limited genetic tools available. Current results suggest an interplay between nutrients, resistance determinants and the end product. Even if many factors and proteins have been found to control A40926 biosynthesis (Figure 4), further studies are necessary to fill the many gaps present in our understanding of the strain’s physiology and of the interplay between A40926 production and resistance before this information can be applied for A40926 yield improvement.

The recent availability of the *N. gerenzanensis* genome sequence [44] and of a large insert library [45] represent important assets for further work on the complex but intriguing regulatory network of the A40926-producing strain. 

## Figures and Tables

**Table 1:** Transcriptional units and biosynthetic roles of the corresponding proteins.

Transcriptional Unit	Function(s)
*dbv1*-*dbv2*	Hpg biosynthesis
*dbv3*	Regulation
*dbv4*	Regulation
*dbv5*-*dbv7*	Hpg biosynthesis; regulation; resistance
*dbv8*-*dbv14*	*N*-Acylation; Halogenation; glycosylation; cross-links
*dbv15*-*dbv17*	NRPS
*dbv18*-*dbv19*	Export
*dbv20*-*dbv21*	Mannose addition; *N*-sugar deacetylation
*dbv22*-*dbv23*	Regulation; mannose *O*-acetylation
*dbv24*-*dbv28*	Export; NRPS; Tyr β-hydroxylation
*dbv29*	*N*-sugar oxidation
*dbv30*-*dbv35*	Dpg biosynthesis
*dbv36*	NRPS accessory protein
*dbv37*	Hpg and Dpg biosynthesis
